# Vagus nerve–driven microbiota homeostasis: a promising integrative add-on therapy for neurodevelopmental disorders

**DOI:** 10.3389/fnins.2026.1855953

**Published:** 2026-07-23

**Authors:** Eric Azabou, Pierre-Yves Frouin, Marc Bellaïche, Irina Duff, Guillaume Bao, Amelie Pillot, Sara Mehlal, Jean Bergounioux, Peter S. Staats, Claire-Marie Rangon

**Affiliations:** 1 SMART-VNS Platform (Structured Multidisciplinary Platform to Advance Research on Therapy of Vagus Nerve Stimulation), Raymond Poincaré Hospital, AP-HP GHU Paris Saclay, Garches, France; 2 Clinical Neurophysiology and Neuromodulation Unit, Department of Physiology, Raymond Poincaré Hospital, AP-HP GHU University Paris Saclay, Garches, France; 3 Inserm UMR 1173 Infection and Inflammation (2I), University of Versailles Saint-Quentin en Yvelines (UVSQ), Paris-Saclay University, Garches, France; 4 Pediatric Gastroenterology Department, Robert Debré Hospital - AP-HP, Paris, France; 5 Neurosurgery Department, Johns Hopkins University, Baltimore, MD, United States; 6 Pediatric Neurology and ICU, Raymond Poincaré Hospital, AP-HP GHU Paris Saclay, Garches, France; 7 National Spine and Pain Centers, Atlantic Beach, FL, United States

**Keywords:** ADHD (attention deficit and hyperactivity disorder), ASD, microbiota-gut-brain axis (MGBA), neurodevelopmental disorders (NDD), nVNS, taVNS, vagus nerve stimulation or VNS

## Abstract

Over the past two decades, the conceptualization of neurodevelopmental disorders (NDDs) has undergone a profound transformation, shifting from a primarily brain-centric framework toward a systems-level perspective that integrates peripheral physiological processes. Among these, the gut microbiota has emerged as a critical determinant of neurodevelopmental trajectories. Through its involvement in immune modulation, metabolic signaling, and neural communication, the microbiota–gut–brain axis (MGBA) exerts a pervasive influence on brain maturation and function. A growing body of evidence indicates that early-life disruptions of microbiota composition—commonly referred to as dysbiosis—are associated with an increased risk of NDDs, including autism spectrum disorder (ASD), attention-deficit/hyperactivity disorder (ADHD), and epilepsy. These disruptions are frequently driven by environmental exposures such as antibiotic use, cesarean delivery, dietary patterns, and psychosocial stress. Despite the expanding recognition of these associations, current therapeutic strategies aimed at restoring microbiota balance, including probiotics and fecal microbiota transplantation, have yielded inconsistent and often transient results. In this context, Vagus Nerve Stimulation (VNS), particularly in its non-invasive forms (nVNS), has emerged as a promising approach capable of modulating environmental exposures through the host-centered regulatory mechanisms of the MGBA. By influencing autonomic tone, activating the cholinergic anti-inflammatory pathway, and modulating neurotransmitter systems, nVNS may restore microbiota homeostasis while simultaneously improving neurodevelopmental outcomes. This article provides a comprehensive and integrative review of the mechanistic, preclinical, and clinical evidence supporting the role of nVNS as a microbiota-modulating intervention. We further discuss its potential as a safe, cost-effective and promising therapeutic strategy for neurodevelopmental disorders, with a particular emphasis on pediatric populations.

## 1 Introduction

Neurodevelopmental disorders (NDDs) constitute a heterogeneous group of conditions characterized by impairments in motor, cognitive, behavioral, and social functioning, with onset typically occurring during early childhood. NDDs are highly prevalent in pediatric populations and encompass a broad range of conditions, including epilepsy, Down syndrome (DS), attention deficit hyperactivity disorder (ADHD), autism spectrum disorder (ASD), Cerebral Palsy (CP), Fetal Alcohol Spectrum Disorder (FASD), Fragile X syndrome (FXS), Tourette syndrome (TS), Rett syndrome (RTT), Prader-Willi syndrome (PWS), Congenital Zika Syndrome (CZS), and Wilson’s Disease (WD). These disorders affect an estimated 10–15% of children worldwide and represent a leading cause of lifelong disability and societal burden (Lord et al., 2020; Thapar and Cooper, 2016).

Historically, the classification of NDDs has progressively shifted from a functional symptomatic-based framework to a more etiological paradigm that emphasizes underlying biologic mechanisms rather than behavioral phenotypes (Ismail and Shapiro, 2019). A unifying etiological framework of NDDS has gradually emerged, proposing that these disorders exist along a continuum rooted in shared biological mechanisms, (Morris-Rosendahl and Crocq, 2020), based upon a common cornerstone, has gradually emerged. As genetic and central nervous system–centric models could not fully account for the complexity and heterogeneity observed across clinical phenotypes, increasing attention has been drawn to the role of environmental and systemic factors in shaping neurodevelopmental outcomes.

Among these, the gut microbiota has emerged as a critical regulator of neurodevelopment (Borre et al., 2014; Parolisi et al., 2023; Wang et al., 2023). The human gastrointestinal tract harbors a vast and dynamic community of microorganisms, collectively referred to as the microbiome, which plays a fundamental role in host physiology. The microbiota is involved in a wide range of processes, including nutrient metabolism, immune system development, and the synthesis of bioactive compounds capable of influencing brain function (Cryan et al., 2019). Dysbiosis, defined as an imbalance in the composition and function of the gut microbiota, has been increasingly implicated in the pathogenesis of neurodevelopmental disorders. This imbalance may involve reduced microbial diversity, loss of beneficial commensal species, or overgrowth of potentially pathogenic organisms. Disruptions of this finely tuned system during this critical developmental window may exert disproportionate effects on neural development, with lasting consequences, contributing to the emergence of NDDs. Consistent with this hypothesis, alterations in gut microbiota composition have been identified across a range of neurodevelopmental disorders (Kang et al., 2013; Strati et al., 2017; Damiani et al., 2023; Charitos et al., 2024). Clinical studies have similarly linked early-life microbiota perturbations—such as those induced by antibiotic exposure—to an increased risk of NDDs (Slykerman et al., 2017). Accumulating evidence supports a potential causal relationship. Longitudinal data, including a large Swedish cohort study following 16,440 children over two decades, support an association between early-life microbial perturbations and later NDD risk (Ahrens et al., 2024). Complementary mechanistic insights are provided by preclinical studies demonstrating that transplantation of gut microbiota from individuals with autism spectrum disorder into germ-free mice can recapitulate behavioral and neurobiological features of the condition (Sharon et al., 2019).

This rational supports the development of gut microbiota-targeted therapeutic strategies in early-life aimed at preventing or mitigating both dysbiosis and neurodevelopmental disorders. Current microbiota focused interventions largely center on dietary optimization, prebiotics, probiotics or synbiotics supplementation, fecal microbiota transplantation (FMT) (Alkuwaiti et al., 2025; Mamun et al., 2025; Marano et al., 2025), phage therapy (addition of bacteriophages to the human microbiome to target the elimination of harmful pathogens without disturbing the beneficial microbial communities) (Talat et al., 2025), CRISPR/Cas9 gene editing (Bell et al., 2017) or mitochondria-focused strategies. A recent systematic review and meta-analysis, assessing the impact of microbiota-based interventions—including synbiotics, prebiotics, single-strain probiotics, probiotic blends, and fecal microbiota transplantation (FMT)—on behavioral outcomes in individuals with ASD, found promising preliminary findings (FMT and probiotic blends particularly) (Hassib et al., 2025). However, several limitations prevented from concluding to clinically validated interventions, just like a former systematic review, published by another team (Tan et al., 2021). In this way, the relatively small number of available studies within specific intervention categories and the heterogeneity across studies, due to inter-individual differences and methodological variabilities, limit the generalizability of the results in microbiome research, no matter the statistical strategies adopted to mitigate these differences (Hassib et al., 2025).

These limitations have prompted interest in alternative approaches that act upstream of the microbiota, targeting the host regulatory systems that govern microbiota composition and function. In this regard, the vagus nerve represents a particularly compelling target (Fülling et al., 2019). Indeed, the available evidence supports the existence of a tightly integrated system in which the vagus nerve and the gut microbiota interact dynamically to regulate neurodevelopmental processes. Interestingly, implanted VNS shares key mechanistic properties with the two different modalities of nVNS, transcutaneous cervical VNS (tVNS) and transcutaneous auricular VNS (taVNS) (Badran et al., 2018; Ingram et al., 2026; Jiakai et al., 2023; Owens et al., 2024; Yue et al., 2023), while offering a favorable safety profile (Aljuhani et al., 2022; Jenkins et al., 2021, 2023). In this context, nVNS has emerged as a promising approach capable of modulating the microbiota through the Microbiota-Gut-Brain Axis (MGBA).

## 2 The microbiota–gut–brain axis: mechanisms and developmental dynamics

The MGBA is a complex bidirectional network that integrates signals between the gastrointestinal tract and the central nervous system. The concept of MGBA comprises interconnected communication pathways involving neural, immune, endocrine, and metabolic mechanisms (Carabotti et al., 2015), each involved in the regulation of neurodevelopmental processes. Importantly, the MGBA is particularly dynamic during early life, a period during which both the microbiota and the brain undergo rapid and coordinated development (Cowan et al., 2020).

### 2.1 Immune pathways

The immune system represents a critical component of the MGBA. The gut microbiota plays a fundamental role in shaping immune development, influencing the balance between pro-inflammatory and anti-inflammatory responses (Sharma et al., 2025). Microbial signals regulate the production of cytokines and chemokines, which can cross the blood–brain barrier or signal through neural pathways to affect brain function.

Microglia, the resident immune cells of the central nervous system, are particularly sensitive to microbial signals. Studies have shown that germ-free animals exhibit immature microglial phenotypes, which can be normalized following colonization with a complex microbiota (Erny et al., 2015). These findings highlight the importance of microbiota-derived signals in the maturation and function of the brain’s immune system (Erny and Prinz, 2020).

### 2.2 Endocrine pathways

The hypothalamic–pituitary–adrenal (HPA) axis constitutes the primary endocrine pathway linking the gut and the brain. Microbial signals can influence the activity of this axis, modulating stress responses and cortisol secretion. Dysregulation of the HPA axis has been implicated in a wide range of neurodevelopmental and psychiatric disorders, including ASD and ADHD (Bertollo et al., 2025; Foster and McVey Neufeld, 2013).

### 2.3 Metabolic pathways

Gut microbiota produce a wide array of metabolites that can influence brain function. Among these, short-chain fatty acids (SCFAs), such as acetate, propionate, and butyrate, have received considerable attention. SCFAs can cross the blood–brain barrier and have been shown to regulate neuroinflammation, synaptic plasticity, and gene expression (Dalile et al., 2019).

### 2.4 Neural pathways: the vagus nerve, a system-level modulator

The vagus nerve serves as the principal neural conduit of the MGBA, providing the most direct pathway for bidirectional communication between the gut and the brain. As the tenth cranial nerve, it provides extensive innervation of the gastrointestinal tract and serves as the primary conduit for afferent sensory signals from the gut to the brain. Approximately 80% of vagal fibers are afferent, transmitting information related to gut physiology, microbial activity, and inflammatory status to the nucleus tractus solitarius (NTS) in the brainstem (Bonaz et al., 2018). From the NTS, signals are relayed to higher-order brain regions, including the hypothalamus, amygdala, and prefrontal cortex, which are involved in emotional regulation, stress responses, and cognitive function (Forstenpointner et al., 2022). This neural pathway enables rapid communication between the gut microbiota and the brain, allowing microbial signals to influence behavior and neurodevelopment in real time.

Growing evidence supports the role of vagus nerve stimulation as a modulator of the MGBA. Indeed, while the field remains relatively recent and heterogeneous, the convergence of preclinical and clinical findings provides a coherent and biologically plausible framework.

## 3 Evidence linking vagus nerve stimulation, microbiota modulation, and neurodevelopment

### 3.1 Experimental evidence: preclinical models establish a causality link between vagus nerve activity, microbiota composition and brain functioning

The literature reveals a striking convergence across experimental paradigms. Preclinical studies allow controlled manipulation of variables and detailed mechanistic investigation. Thus, these models provide a crucial foundation for understanding the causal relationships between vagal activity, microbiota composition and neurodevelopmental outcomes.

Initially, reductions in circulating inflammatory markers following VNS were associated with a shift toward a more balanced microbial ecosystem, given the well-established bidirectional relationship between inflammation and dysbiosis (Pavlov and Tracey, 2012). Thereafter, animal studies have demonstrated that VNS can restore microbial diversity following experimentally induced dysbiosis. In models of inflammatory bowel disease, nVNS/VNS has been shown to increase the abundance of beneficial bacterial taxa while reducing pathogenic species. These changes are accompanied by improvements in intestinal inflammation and barrier function (Quan and Zhou, 2026). Such findings are particularly relevant in the context of neurodevelopmental disorders, where reduced microbial diversity is a common feature. Preclinical studies have shown not only that taVNS shares neurophysiological and biological mechanistic properties with implanted VNS (Badran et al., 2018; Owens et al., 2024; Yue et al., 2023) but also that taVNS is likely to be as effective in a broad range of disorders (Jiakai et al., 2023), notably in ASD (Calikusu et al., 2026), without adverse effects [largely limited to implantable devices (Ben-Menachem, 2001)]. By promoting a more diverse and stable microbiota, nVNS may help restore key metabolic and immunological functions.

Subsequently, several studies have demonstrated that the behavioral effects of microbiota manipulation are dependent on an intact vagus nerve. For instance, administration of specific probiotic strains can reduce anxiety-like behavior in rodents, but these effects are abolished following vagotomy (Bravo et al., 2011). This suggests that the vagus nerve is a critical mediator of microbiota-induced behavioral changes. Similarly, germ-free animals colonized with microbiota from diseased donors induced either both structural abnormalities of the vagal nerve and behavioral abnormalities (Kim et al., 2020) or brain lesions (hippocampus) that could be modulated remarkably by pharmaco-genetic interventions targeting vagal signaling (Rei et al., 2022). These findings highlight the central role of the vagus nerve in translating microbial signals into behavioral outcomes.

While most mechanistic insights are derived from preclinical studies, an increasing number of clinical investigations have begun to explore the effects of nVNS on the MGBA in humans.

### 3.2 Human evidence: emerging clinical data support an association between vagus nerve activity, microbiota composition and brain functioning

Although still limited, early clinical studies also suggest that nVNS may have beneficial effects in neurodevelopmental disorders, given its ability to influence multiple physiological systems, including immune regulation, autonomic balance, and gastrointestinal function. Indeed, nVNS can reduce circulating inflammatory markers (Lerman et al., 2016), including tumor necrosis factor (TNF) and interleukin-6 (IL-6) (Huguenard et al., 2025) and improve autonomic balance (as evidenced by changes in heart rate variability) (Machetanz et al., 2021), both of which are closely linked to microbiota homeostasis (Bonaz et al., 2018; Quan and Zhou, 2026). Besides, increased vagal tone is associated with enhanced resilience to stress since early life (Marvin et al., 2019). These properties make it particularly relevant in the context of disorders characterized by dysregulation of the MGBA.

Epilepsy represents the most well-established clinical indication for VNS and provides a valuable framework for understanding its potential mechanisms in other neurodevelopmental conditions. Since its approval in the late 1990s, VNS has demonstrated efficacy in reducing seizure frequency in patients with refractory epilepsy, with response rates ranging from 30% to 50% depending on patient populations (Ben-Menachem, 2002; Groves and Brown, 2005). Importantly, the benefits of VNS in epilepsy extend beyond seizure control. Numerous studies have reported improvements in mood, cognitive function, and quality of life, suggesting that VNS exerts widespread effects on brain function (Yuan and Silberstein, 2017). These observations are consistent with its influence on central neurotransmitter systems, including the locus coeruleus–noradrenergic and dorsal raphe–serotonergic pathways (Manta et al., 2013). From the perspective of the MGBA, epilepsy is increasingly recognized as a disorder involving systemic inflammation and microbiota alterations. Recent studies have demonstrated differences in gut microbiota composition between epileptic patients and healthy controls, as well as changes associated with ketogenic diet therapy (Olson et al., 2018). In this context, nVNS may exert part of its therapeutic effect through modulation of gut–brain interactions, although direct evidence remains limited.

Case series and small pilot studies have reported improvements in social interaction, communication, and emotional regulation following VNS, particularly in ASD patients with comorbid epilepsy (Levy et al., 2010; Van Hoorn et al., 2019; Wang et al., 2022). While these studies are limited by small sample sizes or lack of control groups, they have provided a proof of concept for the therapeutic potential of vagal modulation in ASD, given the strong and reproducible association between ASD and gut microbiota alterations (Andreo-Martínez et al., 2022; Iglesias-Vázquez et al., 2020; Lewandowska-Pietruszka et al., 2023; Ma et al., 2019; Pulikkan et al., 2018; Wang et al., 2014; Yang et al., 2024), as well as parasympathetic under-activation (Bufo et al., 2025; Kalfiřt et al., 2023) and a high prevalence of gastrointestinal symptoms (up to 70%), often correlated with behavioral severity in patients with ASD (Mayer et al., 2015). A preliminary study exploring the use of taVNS in ASD have yielded encouraging results, reporting improvements in social behavior and emotional regulation (Black et al., 2023), inciting new trials (Sun et al., 2024). While these findings require confirmation in larger randomized trials, they are consistent with the hypothesis that enhancing vagal tone can restore balance within the MGBA, linking vagal activity to microbiota and brain function.

Attention-deficit/hyperactivity disorder is another condition in which dysregulation of autonomic function and inflammation has been implicated. Although the role of the microbiota in ADHD is less well established than in ASD (Bundgaard-Nielsen et al., 2023; Gkougka et al., 2022; Sukmajaya et al., 2021), emerging evidence suggests that alterations in microbial composition and metabolic function may contribute to symptomatology (Aarts et al., 2017; Novau-Ferré et al., 2025). The application of nVNS in ADHD remains largely exploration (Luna et al., 2025). However, given its effects on noradrenergic and dopaminergic systems—both of which are central to ADHD pathophysiology—nVNS represents a plausible therapeutic approach (Zaehle and Krauel, 2021; Zhi et al., 2024). Moreover, its capacity to modulate stress responses (Kim et al., 2025) and autonomic balance (De Moraes et al., 2023) may address underlying vulnerabilities that contribute to attentional and behavioral dysregulation.

Beyond ASD and ADHD, nVNS has been investigated in a range of conditions characterized by dysregulation of the MGBA, including anxiety disorders, depression, and post-traumatic stress disorder (Tyler, 2025). While these conditions are not strictly classified as neurodevelopmental disorders, they share common mechanistic pathways, including inflammation, altered neurotransmission, and microbiota perturbations. In pediatric populations, the potential applications of nVNS also extend to conditions such as developmental delay and genetic syndromes associated with autonomic dysfunction like Prader-Willi Syndrome (Manning et al., 2016; Schmausser et al., 2024). However, robust clinical data in these populations remain limited, highlighting the need for well-designed prospective studies.

To sum up, importantly, direct microbiota sequencing data in human nVNS studies remain limited (Liu et al., 2024). Mostly indirect evidence supports a therapeutic modulatory role for nVNS in NDDs, so far. Nevertheless, a recent systematic review has positioned VNS/nVNS as an “emerging paradigm for gut microbiota modulation” (Liang et al., 2026).

### 3.3 Toward an integrative mechanistic model of vagus-mediated microbiota regulation

**Figure 1:**
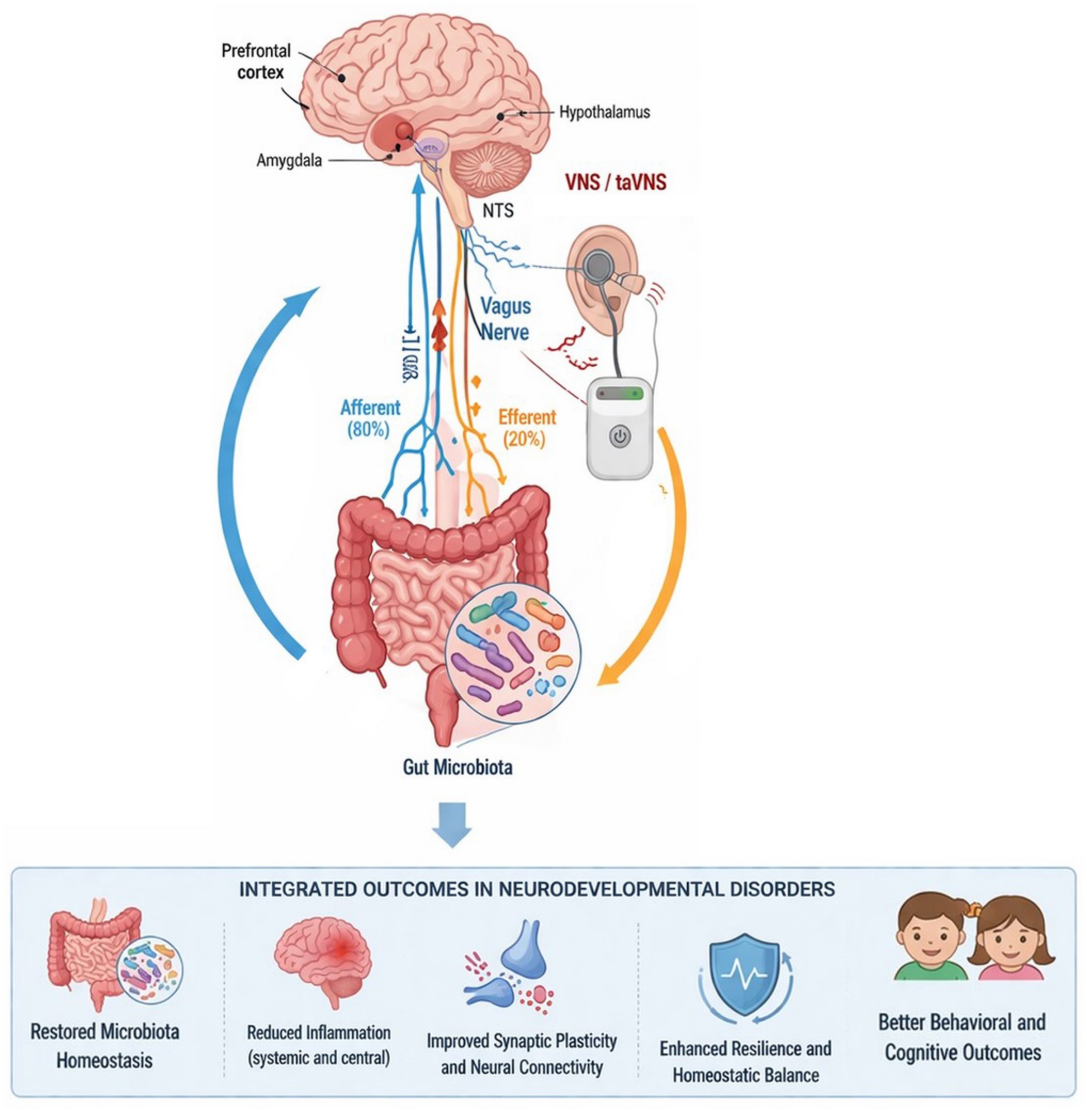
Mechanisms of Action of transcutaneous auricular Vagus Nerve Stimulation in Neurodevelopmental Disorders.

The convergence of evidence from preclinical and clinical studies supports the development of an integrative model in which the vagus nerve acts as a central regulator of the MGBA. This model emphasizes the bidirectional and dynamic nature of interactions between the gut microbiota and the brain, mediated through neural, immune, endocrine, and metabolic pathways (see Table 1; Figure 1).

**Table 1:** Putative mechanisms of action of vagus nerve stimulation in neurodevelopmental disorders.

Actions of VNS/taVNS	Level/axis	Main components	Effects on brain/neurodevelopment	Emerging preclinical studies	Established preclinical studies	Emerging clinical trials
Vagal tone enhancement and Gut–brain signal transmission improvement	**Neural** (afferent pathway)	Nucleus Tractus Solitarius Locus Caeruleus Raphe nuclei Hypothalamus, Amygdala, Prefrontal cortex Serotonin, dopamine, norepinephrine, GABA	Rapid regulation of Stress response mood, social behavior, attention and cognition	Calikusu et al. (2026), Minaya et al. (2024), Bravo et al. (2011)	Owens et al. (2024), Kim et al. (2020), Kaelberer et al. (2018)	Tyler (2025), Bufo et al. (2025), Schmausser et al. (2024), Black et al. (2023), Kalfiřt et al. (2023), Jenkins et al. (2023), Wang et al. (2022), Machetanz et al. (2021), Manning et al. (2016)
Gastrointestinal homeostasis restoration and dysbiosis reduction	**Neural** (efferent pathway)	Enterocytes, Tight junction proteins, Enteric nervous system Inflammatory mediators Microbiota composition	Regulation of gut motility, secretion, intestinal barrier integrity Reduced systemic immune activation	Liang et al. (2026)	Wang et al. (2024), Liu et al. (2024)	Quan and Zhou (2026), Jenkins et al. (2023), Jenkins et al. (2021)
Decrease of systemic inflammation	**Immune** Cholinergic Anti-inflammatory Reflex Microglia maturation	α7-nAcetylCholine Receptors, macrophages, TNF, IL-6 Microbiota-derived signals Microglia	Reduction of systemic and neuroinflammation, Protection of developing neural circuits Proper synaptic pruning and neurodevelopmental maturation	Frasch et al. (2016), Bonaz et al. (2016), Tracey (2002)	Zhao et al. (2025)	Quan and Zhou (2026), Huguenard et al. (2025), Lerman et al. (2016)
Stress responses stabilization	**Endocrine**	Hypothalamic–pituitary–adrenal axis	Modulation of anxiety, stress reactivity, attention and behavior	Bonaz et al. (2018)	Yue et al. (2023)	Kim et al. (2025), Luna et al. (2025)
Indirect modulation via restoration of gut ecosystem balance	**Metabolic**	Short-chain fatty acids (acetate, propionate, butyrate)	Regulation of gene expression, neuroinflammation, and synaptic plasticity	Dalile et al. (2019)	Khuanjing et al. (2023), Xue et al. (2017)	Quan and Zhou (2026), De Moraes et al. (2023)
Bottom-Up and Top-Down regulator	**Dynamic Bidirectional Loop**	Integrated neural–immune–endocrine–metabolic network	Maintenance of Neurodevelopmental Homeostasis	Sanders et al. (2019)	Rei et al. (2022)	Badran et al. (2018)

The vagus nerve is uniquely positioned to detect microbial-derived signals within the gut. Firstly, via enteroendocrine cells, interspersed throughout the intestinal epithelium, acting as key intermediaries in this process. These cells respond to microbial metabolites—such as short-chain fatty acids (SCFAs), bile acids, and tryptophan derivatives—by releasing neurotransmitters and hormones that activate vagal afferents (Kaelberer et al., 2018). This mechanism allows for rapid transmission of information from the gut to the brain, effectively translating microbial activity into neural signals. For example, specific strains of Lactobacillus have been shown to modulate GABA receptor expression in the brain via vagal pathways, an effect that is abolished following vagotomy (Bravo et al., 2011). Such findings underscore the essential role of the vagus nerve in mediating microbiota-driven effects on brain function. In addition to its afferent functions, the vagus nerve exerts powerful efferent control over gastrointestinal physiology. Through its influence on gut motility, secretion, and barrier function, vagal activity shapes the ecological environment in which microbiota reside.

Secondly, through the maintenance of intestinal barrier integrity. Increased intestinal permeability, often referred to as “leaky gut,” has been implicated in the pathogenesis of several neurodevelopmental disorders, particularly ASD. By enhancing tight junction integrity and reducing inflammation, vagal activation can help restore barrier function (Wang et al., 2024) and limit the translocation of microbial products into the systemic circulation (Bonaz et al., 2016).

Thirdly, a central mechanism through which the vagus nerve influences the microbiota is the cholinergic anti-inflammatory pathway. Activation of this pathway results in the suppression of pro-inflammatory cytokine release via α7 nicotinic acetylcholine receptors expressed on macrophages and other immune cells (Tracey, 2002). Chronic inflammation is a well-established driver of dysbiosis, creating an environment that favors the growth of pathogenic bacteria at the expense of beneficial commensals. By dampening inflammatory responses, nVNS may indirectly promote a more balanced microbiota composition.

Lastly, nVNS also influences the production and release of key neurotransmitters, including norepinephrine, serotonin, and acetylcholine. These neurotransmitters, in turn, can affect microbial growth and metabolism. For example, norepinephrine has been shown to modulate bacterial virulence and biofilm formation, while serotonin can influence gut motility and microbial composition (Mayer et al., 2015). At the same time, microbial metabolites can modulate vagal activity, creating a reciprocal relationship. SCFAs, for instance, have been shown to influence vagal signaling and central nervous system function, highlighting the interconnected nature of these pathways (Dalile et al., 2019).

At the core of this model lies the concept of homeostatic regulation. Under physiological conditions, the microbiota and the host maintain a mutually beneficial relationship characterized by stability and resilience. Disruptions to this system, whether due to environmental factors, genetic predisposition, or disease can lead to a state of dysbiosis, which in turn exacerbates inflammation and neural dysfunction. nVNS can be conceptualized as a bottom-up and top-down regulatory intervention that restores balance across these interconnected systems. By enhancing vagal tone, VNS increases afferent signaling from the gut to the brain, improving the detection and integration of microbial signals. At the same time, efferent vagal pathways modulate gut physiology, immune responses, and barrier function, creating an environment that supports the re-establishment of a healthy microbiota. This dual action distinguishes VNS from conventional microbiota-targeted therapies, which typically focus on altering microbial composition without addressing the underlying regulatory mechanisms. As such, VNS may offer a more durable and systemic approach to restoring microbiota homeostasis.

## 4 Discussion

The present synthesis supports a paradigm shift in the understanding and management of neurodevelopmental disorders, moving from a predominantly brain-centered model toward a systems-level framework integrating the MGBA. Within this framework, the vagus nerve emerges not merely as a passive conduit of information but as a central regulatory node capable of orchestrating bidirectional communication between the gut microbiota and the central nervous system.

One of the most compelling aspects of this model is its ability to reconcile disparate observations across multiple domains. For instance, the coexistence of gastrointestinal symptoms, immune dysregulation, and behavioral abnormalities in conditions such as ASD can be understood as manifestations of a common underlying disruption of MGBA homeostasis (Cryan et al., 2019; Morais et al., 2021). Moreover, and importantly, while microbiota alterations are consistently observed across NDDs, their specific profiles often differ between conditions (Bundgaard-Nielsen et al., 2023; Hokanson et al., 2024), reflecting a heterogeneous yet convergent pattern of dysregulation. Rather than attempting to correct individual microbial imbalances in a disorder-specific manner, a more integrative approach may be to restore global microbiota homeostasis. Similarly, the beneficial effects of interventions as diverse as dietary modification, probiotics, and nVNS may converge on shared mechanisms involving inflammation, barrier integrity, and neural signaling. Remarkably, several microbiota-targeted therapies appear to depend, at least in part, on intact vagal signaling pathways—such as fecal microbiota transplantation and probiotic interventions (Bravo et al., 2011; Minaya et al., 2024; Mörkl et al., 2025, 20) or may be potentiated by vagal activation, as suggested in mitochondria-focused strategies (Arthur et al., 2007; Khuanjing et al., 2023; Kim et al., 2022; Xue et al., 2017; Zhao et al., 2025). Last, the gut microbiota is increasingly exposed to widespread environmental disruptions. Prolonged dysbiosis may result from rising rates of cesarean delivery (Thomaidi et al., 2025), high intake of ultra-processed foods (Spiller et al., 2025), stress (Zhang et al., 2024) and early-life antibiotic exposure or other commonly prescribed medication. Antibiotics remain a major and often unavoidable driver of gut microbiota disruption (Ramirez et al., 2020). In New Zealand, 97% of children had received at least one course of antibiotics by the age of 5 years (Hobbs et al., 2017). Children in low-income settings are also commonly exposed to antibiotics, with an average consumption of 4.9 courses per child-year reported among children aged <2 years in eight low-income settings (Bangladesh, Brazil, India, Nepal, Pakistan, Peru, South Africa and Tanzania) year was reported among children aged <2 years. A key strength of nVNS as a therapeutic approach lies in its systems-level mode of action, probably through epigenomic reprogramming (Sanders et al., 2019), as gut microbiota itself (Rubas et al., 2025). Therefore, unlike interventions that target individual microbial species or metabolic pathways, nVNS acts on the regulatory networks that govern the entire ecosystem. This may explain its potential to produce more durable and generalized effects.

Another important consideration is the bidirectional nature of the MGBA. While much attention has been devoted to the influence of microbiota on brain function, it is increasingly clear that the brain, through the autonomic nervous system, plays an equally important role in shaping the microbiota. Stress, for example, can alter gut motility, secretion, and immune function, leading to changes in microbial composition (Foster and McVey Neufeld, 2013). By counteracting these effects, nVNS may help break the vicious cycle linking stress, dysbiosis, and neurodevelopmental dysfunction. From a translational perspective, the use nVNS represents a particularly promising avenue. Techniques such as taVNS allow for non-invasive, repeated stimulation without the need for surgical implantation. This greatly expands the potential applicability of nVNS, particularly in pediatric populations and in preventive settings.

However, despite these promising features, several challenges must be addressed before nVNS can be fully integrated into clinical practice for NDDs. The current body of evidence, while encouraging, remains limited in several important respects. First, direct evidence linking nVNS to changes in human gut microbiota composition is still scarce. Most clinical studies have focused on indirect markers, such as inflammatory cytokines or autonomic function, rather than performing detailed microbiome analyses. As a result, the extent to which nVNS directly alters microbial ecosystems in humans remains to be fully elucidated. Second, the available clinical studies are often characterized by small sample sizes, heterogeneity of populations, and lack of randomized controlled designs. This limits the generalizability of findings and underscores the need for larger, well-designed trials. Third, there is currently no standardized protocol for nVNS in the context of neurodevelopmental disorders. Questions regarding optimal timing (night versus daytime), dosing (parameters such as stimulation frequency, intensity, duration, treatment schedule) and the choice of the neuromodulation placebo must be addressed through carefully designed clinical trials. Fourth, while non-invasive techniques are generally considered safe, long-term safety data in pediatric populations are limited. Indeed, early life represents a critical window for both microbiota maturation and brain development and must be handled with caution since the long-term effects of nVNS in infants remain insufficiently studied. It is noteworthy that no dedicated pediatric nVNS device exist (Sigrist et al., 2023) and that nVNS devices are not currently approved under the age of three.

Nevertheless, non-invasive VNS techniques like taVNS are especially well suited for pediatric and neonatal use, given their favorable short-term safety and ease of administration. Several studies have shown that non-invasive or minimally invasive VNS devices are safe in neonates (Aljuhani et al., 2022; Badran et al., 2020; Jenkins et al., 2021, 2023) and allow strong positive clinical outcomes in randomized, blinded, healthy control trials (Lerman et al., 2016). taVNS has begun to be assessed in children, from 7 to 16 years-old, with ASD (Black et al., 2023). Consequently, although nVNS is still insufficiently validated clinically and is not a near-clinical solution for NDDs yet, research protocols and clinical trials assessing its impact on maladaptive neural circuits during critical periods of brain development are fully justified, from an ethical point of view (Abarca-Castro et al., 2023; Gargus et al., 2026). Preclinical invasive VNS use during gestation has even turned out to be safe and beneficial in the near-term fetal sheep model (Cao et al., 2024; Wakefield et al., 2025). Thereby, this model of human fetal development, due to the unique amenability of the unanesthetized fetal sheep to the surgical placement and maintenance of catheters and electrodes (allowing notably repetitive application of electric stimulation), supports that early invasive vagus nerve stimulation may be both safe and effective for modifying disease trajectories (Frasch et al., 2016, 2018). Recently, a neonatal rat model using taVNS was also validated as an animal model of non-invasive taVNS for preterm infants (Gail et al., 2023).

Future research should aim to address these limitations through a combination of mechanistic, clinical, and translational studies. One priority is the integration of microbiome sequencing into clinical trials of nVNS. By directly assessing changes in microbial composition and function, researchers can better understand the mechanisms underlying nVNS effects and identify biomarkers of response. Another important avenue is the development of personalized approaches to nVNS therapy. Given the heterogeneity of both microbiome and neurodevelopmental disorders, it is unlikely that a one-size-fits-all approach will be optimal. Instead, treatment protocols may need to be tailored based on individual characteristics, including microbiota profiles, genetic background, and clinical phenotype. The combination of nVNS with other microbiota-targeted interventions also represents a promising strategy. For example, synergistic effects may be achieved by combining nVNS with dietary interventions, probiotics, or prebiotics, thereby addressing both the regulatory and compositional aspects of the microbiota. Finally, there is a need to explore the preventive potential of nVNS. Early identification of at-risk individuals, followed by targeted interventions during critical developmental windows, could potentially alter disease trajectories and reduce the burden of neurodevelopmental disorders.

The increasing prevalence of neurodevelopmental disorders, combined with the limitations of existing therapeutic approaches, underscores the need for innovative interventions. In this context, VNS particularly in its non-invasive forms, offers several translational advantages regarding public health perspectives. First, it is inherently cost-effective compared to pharmacological therapies and invasive procedures. Second, it has the potential for widespread deployment in both high- and low-resource settings. Third, it is multimodal, targeting multiple pathophysiological pathways simultaneously. Thus, from a public health perspective, nVNS represents a promising integrative adjunctive therapy for neurodevelopmental disorders.

## 5 Conclusion

In conclusion, neurodevelopmental disorders represent one of the most pressing and underserved challenges in modern medicine. While historically approached from a symptom-based perspective, a shift toward mechanism-driven strategies offers new therapeutic options. As such, the integration of the microbiota–gut–brain axis into the conceptual framework of neurodevelopmental disorders may represent a major advance in our understanding of these conditions. Within this framework, the vagus nerve emerges as a central regulator capable of modulating multiple interconnected systems.

Hence, non-invasive vagus nerve stimulation offers a novel and promising approach to restoring microbiota homeostasis and improving neurodevelopmental outcomes. By acting on the regulatory networks that govern the MGBA, nVNS provides a system-level intervention that addresses the underlying mechanisms of dysbiosis and neurodevelopmental dysfunction.

While significant challenges remain, the convergence of mechanistic, preclinical, and clinical evidence supports the continued investigation of nVNS as a therapeutic and preventive strategy. The convenience, cost effectiveness and safety of nVNS should make it a prime target for financial and institutional commitment to launch prospective, controlled large-scale clinical trials including microbiome-integrated research in pediatric populations. With further mechanistic and clinical validation, nVNS has the potential to become a key component of a new generation of systems-based interventions targeting the microbiota–gut–brain axis and promoting healthy neurodevelopment.

## Data Availability

The original contributions presented in the study are included in the article/supplementary material, further inquiries can be directed to the corresponding author.

## References

[ref1] Aarts E. Ederveen T. H. A. Naaijen J. Zwiers M. P. Boekhorst J. Timmerman H. M. . (2017). Gut microbiome in ADHD and its relation to neural reward anticipation. PLoS One 12:e0183509. doi: 10.1371/journal.pone.0183509, 28863139 PMC5581161

[ref2] Abarca-Castro E. A. Talavera-Peña A. K. Reyes-Lagos J. J. Becerril-Villanueva E. Pérez-Sanchez G. De La Peña F. R. . (2023). Modulation of vagal activity may help reduce neurodevelopmental damage in the offspring of mothers with pre-eclampsia. Front. Immunol. 14:1280334. doi: 10.3389/fimmu.2023.1280334, 38022681 PMC10653300

[ref3] Ahrens A. P. Hyötyläinen T. Petrone J. R. Igelström K. George C. D. Garrett T. J. . (2024). Infant microbes and metabolites point to childhood neurodevelopmental disorders. Cell 187, 1853–1873.e15. doi: 10.1016/j.cell.2024.02.035, 38574728

[ref4] Aljuhani T. Haskin H. Davis S. Reiner A. Moss H. G. Badran B. W. . (2022). Transcutaneous auricular vagus nerve stimulation (taVNS) given for poor feeding in at-risk infants also improves their motor abilities. J. Pediatr. Rehabil. Med. 15, 447–457. doi: 10.3233/PRM-210090, 36093716 PMC9976577

[ref5] Alkuwaiti S. H. Skrabulyte-Barbulescu J. Yassin L. K. Almazrouei S. Aldhaheri D. Alderei M. . (2025). Harnessing the microbiota-gut–brain axis to prevent and treat pediatric neurodevelopmental disorders: translational insights and strategies. J. Transl. Med. 23:1286. doi: 10.1186/s12967-025-07279-4, 41239321 PMC12619268

[ref6] Andreo-Martínez P. Rubio-Aparicio M. Sánchez-Meca J. Veas A. Martínez-González A. E. (2022). A Meta-analysis of gut microbiota in children with autism. J. Autism Dev. Disord. 52, 1374–1387. doi: 10.1007/s10803-021-05002-y, 33948825

[ref7] Arthur T. M. Saneto R. P. De Menezes M. S. Devinsky O. LaJoie J. Murphy P. J. . (2007). Vagus nerve stimulation in children with mitochondrial electron transport chain deficiencies. Mitochondrion 7, 279–283. doi: 10.1016/j.mito.2007.04.003, 17513178

[ref8] Badran B. W. Dowdle L. T. Mithoefer O. J. LaBate N. T. Coatsworth J. Brown J. C. . (2018). Neurophysiologic effects of transcutaneous auricular vagus nerve stimulation (taVNS) via electrical stimulation of the tragus: a concurrent taVNS/fMRI study and review. Brain Stimul. 11, 492–500. doi: 10.1016/j.brs.2017.12.009, 29361441 PMC6487660

[ref9] Badran B. W. Jenkins D. D. Cook D. Thompson S. Dancy M. DeVries W. H. . (2020). Transcutaneous auricular Vagus nerve stimulation-paired rehabilitation for Oromotor feeding problems in newborns: an open-label pilot study. Front. Hum. Neurosci. 14:77. doi: 10.3389/fnhum.2020.00077, 32256328 PMC7093597

[ref10] Bell S. Peng H. Crapper L. Kolobova I. Maussion G. Vasuta C. . (2017). A rapid pipeline to model rare neurodevelopmental disorders with simultaneous CRISPR/Cas9 gene editing. Stem Cells Transl. Med. 6, 886–896. doi: 10.1002/sctm.16-0158, 28170165 PMC5442775

[ref11] Ben-Menachem E. (2001). Vagus nerve stimulation, side effects, and long-term safety. J. Clin. Neurophysiol. 18, 415–418. doi: 10.1097/00004691-200109000-00005, 11709646

[ref12] Ben-Menachem E. (2002). Vagus-nerve stimulation for the treatment of epilepsy. The Lancet Neurology 1, 477–482. doi: 10.1016/S1474-4422(02)00220-X, 12849332

[ref13] Bertollo A. G. Puntel C. F. Da Silva B. V. Martins M. Bagatini M. D. Ignácio Z. M. (2025). Neurobiological relationships between neurodevelopmental disorders and mood disorders. Brain Sci. 15:307. doi: 10.3390/brainsci15030307, 40149827 PMC11940368

[ref14] Black B. Hunter S. Cottrell H. Dar R. Takahashi N. Ferguson B. J. . (2023). Remotely supervised at-home delivery of taVNS for autism spectrum disorder: feasibility and initial efficacy. Front. Psych. 14:1238328. doi: 10.3389/fpsyt.2023.1238328, 37840787 PMC10568329

[ref15] Bonaz B. Bazin T. Pellissier S. (2018). The Vagus nerve at the Interface of the microbiota-gut-brain Axis. Front. Neurosci. 12:49. doi: 10.3389/fnins.2018.00049, 29467611 PMC5808284

[ref16] Bonaz B. Sinniger V. Pellissier S. (2016). Anti-inflammatory properties of the vagus nerve: potential therapeutic implications of vagus nerve stimulation. J. Physiol. 594, 5781–5790. doi: 10.1113/JP271539, 27059884 PMC5063949

[ref17] Borre Y. E. O’Keeffe G. W. Clarke G. Stanton C. Dinan T. G. Cryan J. F. (2014). Microbiota and neurodevelopmental windows: implications for brain disorders. Trends Mol. Med. 20, 509–518. doi: 10.1016/j.molmed.2014.05.002, 24956966

[ref18] Bravo J. A. Forsythe P. Chew M. V. Escaravage E. Savignac H. M. Dinan T. G. . (2011). Ingestion of Lactobacillus strain regulates emotional behavior and central GABA receptor expression in a mouse via the vagus nerve. Proc. Natl. Acad. Sci. 108, 16050–16055. doi: 10.1073/pnas.1102999108, 21876150 PMC3179073

[ref19] Bufo M. R. Guidotti M. Lemaire M. Malvy J. Houy-Durand E. Bonnet-Brilhault F. . (2025). Autonomic disequilibrium at rest in autistic children and adults. Appl. Psychophysiol. Biofeedback 50, 465–480. doi: 10.1007/s10484-025-09696-z, 39982620 PMC12331820

[ref20] Bundgaard-Nielsen C. Lauritsen M. B. Knudsen J. K. Rold L. S. Larsen M. H. Hindersson P. . (2023). Children and adolescents with attention deficit hyperactivity disorder and autism spectrum disorder share distinct microbiota compositions. Gut Microbes 15:2211923. doi: 10.1080/19490976.2023.2211923, 37199526 PMC10197996

[ref21] Calikusu A. Ince M. S. Bolay H. Atalar K. Yigman Z. Topa E. . (2026). Targeting brain plasticity: vagal nerve stimulation as a therapy for autism-like symptoms in a valproic acid mouse model. Dev. Neurobiol. 86:e23019. doi: 10.1002/dneu.23019, 41273029

[ref22] Cao M. Kuthiala S. Jean K. J. Liu H. L. Courchesne M. Nygard K. . (2024). The Vagus nerve regulates Immunometabolic homeostasis in the ovine fetus near term: the impact on terminal ileum. Biology 13:38. doi: 10.3390/biology13010038, 38248469 PMC10812930

[ref23] Carabotti M. Scirocco A. Maselli M. A. Severi C. (2015). The gut-brain axis: interactions between enteric microbiota, central and enteric nervous systems. Ann. Gastroenterol. 28, 203–209. 25830558 PMC4367209

[ref24] Charitos I. A. Inchingolo A. M. Ferrante L. Inchingolo F. Inchingolo A. D. Castellaneta F. . (2024). The gut microbiota’s role in neurological, psychiatric, and neurodevelopmental disorders. Nutrients 16:4404. doi: 10.3390/nu16244404, 39771025 PMC11677138

[ref25] Cowan C. S. M. Dinan T. G. Cryan J. F. (2020). Annual research review: critical windows – the microbiota–gut–brain axis in neurocognitive development. J. Child Psychol. Psychiatry 61, 353–371. doi: 10.1111/jcpp.13156, 31773737

[ref26] Cryan J. F. O’Riordan K. J. Cowan C. S. M. Sandhu K. V. Bastiaanssen T. F. S. Boehme M. . (2019). The microbiota-gut-brain Axis. Physiol. Rev. 99, 1877–2013. doi: 10.1152/physrev.00018.2018, 31460832

[ref27] Dalile B. Van Oudenhove L. Vervliet B. Verbeke K. (2019). The role of short-chain fatty acids in microbiota–gut–brain communication. Nat. Rev. Gastroenterol. Hepatol. 16, 461–478. doi: 10.1038/s41575-019-0157-3, 31123355

[ref28] Damiani F. Cornuti S. Tognini P. (2023). The gut-brain connection: exploring the influence of the gut microbiota on neuroplasticity and neurodevelopmental disorders. Neuropharmacology 231:109491. doi: 10.1016/j.neuropharm.2023.109491, 36924923

[ref29] De Moraes T. L. Costa F. O. Cabral D. G. Fernandes D. M. Sangaleti C. T. Dalboni M. A. . (2023). Brief periods of transcutaneous auricular vagus nerve stimulation improve autonomic balance and alter circulating monocytes and endothelial cells in patients with metabolic syndrome: a pilot study. Bioelectron. Med. 9:7. doi: 10.1186/s42234-023-00109-2 36998060 PMC10064781

[ref30] Erny D. Hrabě De Angelis A. L. Jaitin D. Wieghofer P. Staszewski O. David E. . (2015). Host microbiota constantly control maturation and function of microglia in the CNS. Nat. Neurosci. 18, 965–977. doi: 10.1038/nn.4030, 26030851 PMC5528863

[ref31] Erny D. Prinz M. (2020). How microbiota shape microglial phenotypes and epigenetics. Glia 68, 1655–1672. doi: 10.1002/glia.23822, 32181523

[ref32] Forstenpointner J. Maallo A. M. S. Elman I. Holmes S. Freeman R. Baron R. . (2022). The solitary nucleus connectivity to key autonomic regions in humans. Eur. J. Neurosci. 56, 3938–3966. doi: 10.1111/ejn.15691, 35545280 PMC12918705

[ref33] Foster J. A. McVey Neufeld K.-A. (2013). Gut–brain axis: how the microbiome influences anxiety and depression. Trends Neurosci. 36, 305–312. doi: 10.1016/j.tins.2013.01.005, 23384445

[ref34] Frasch M. G. Burns P. Benito J. Cortes M. Cao M. Fecteau G. . (2018). “Sculpting the sculptors: methods for studying the fetal cholinergic signaling on systems and cellular scales,” in Psychoneuroimmunology, ed. Yan Q., vol. 1781 (New York: Springer), 341–352. 10.1007/978-1-4939-7828-1_18 29705856

[ref35] Frasch M. G. Szynkaruk M. Prout A. P. Nygard K. Cao M. Veldhuizen R. . (2016). Decreased neuroinflammation correlates to higher vagus nerve activity fluctuations in near-term ovine fetuses: a case for the afferent cholinergic anti-inflammatory pathway? J. Neuroinflammation 13:103. doi: 10.1186/s12974-016-0567-x, 27165310 PMC4894374

[ref36] Fülling C. Dinan T. G. Cryan J. F. (2019). Gut microbe to brain signaling: what happens in Vagus…. Neuron 101, 998–1002. doi: 10.1016/j.neuron.2019.02.008, 30897366

[ref37] Gail M. W. Sims-Robinson C. Boger H. Ergul A. Mukherjee R. Jenkins D. D. . (2023). Transcutaneous auricular vagus nerve stimulation (taVNS) decreases heart rate acutely in neonatal rats. Brain Stimul. 16, 1240–1242. doi: 10.1016/j.brs.2023.08.018, 37619892 PMC10795507

[ref38] Gargus M. Poyhia L. Errico J. P. Rogers M. K. N. Tremblay M.-E. (2026). Vagus nerve stimulation as an anti-inflammatory therapy for maternal immune activation-induced alterations in offspring microglia and neurodevelopment. Front. Neurosci. 20:1827714. doi: 10.3389/fnins.2026.1827714, 42110197 PMC13153084

[ref39] Gkougka D. Mitropoulos K. Tzanakaki G. Panagouli E. Psaltopoulou T. Thomaidis L. . (2022). Gut microbiome and attention deficit/hyperactivity disorder: a systematic review. Pediatr. Res. 92, 1507–1519. doi: 10.1038/s41390-022-02027-6, 35354932

[ref40] Groves D. A. Brown V. J. (2005). Vagal nerve stimulation: a review of its applications and potential mechanisms that mediate its clinical effects. Neurosci. Biobehav. Rev. 29, 493–500. doi: 10.1016/j.neubiorev.2005.01.004, 15820552

[ref41] Hassib L. Kanashiro A. Pedrazzi J. F. C. Vercesi B. F. Higa S. Arruda Í. . (2025). Microbiota-based therapies as novel targets for autism spectrum disorder: a systematic review and meta-analysis. Prog. Neuro-Psychopharmacol. Biol. Psychiatry 139:111385. doi: 10.1016/j.pnpbp.2025.111385, 40348275

[ref42] Hobbs M. R. Grant C. C. Ritchie S. R. Chelimo C. Morton S. M. B. Berry S. . (2017). Antibiotic consumption by New Zealand children: exposure is near universal by the age of 5 years. J. Antimicrob. Chemother. 72, 1832–1840. doi: 10.1093/jac/dkx060, 28333294

[ref43] Hokanson K. C. Hernández C. Deitzler G. E. Gaston J. E. David M. M. (2024). Sex shapes gut–microbiota–brain communication and disease. Trends Microbiol. 32, 151–161. doi: 10.1016/j.tim.2023.08.013, 37813734

[ref44] Huguenard A. L. Tan G. Rivet D. J. Gao F. Johnson G. W. Adamek M. . (2025). Auricular vagus nerve stimulation for mitigation of inflammation and vasospasm in subarachnoid hemorrhage: a single-institution randomized controlled trial. J. Neurosurg. 142, 1720–1731. doi: 10.3171/2024.10.JNS241643, 39854697 PMC12283154

[ref45] Iglesias-Vázquez L. Van Ginkel Riba G. Arija V. Canals J. (2020). Composition of gut microbiota in children with autism Spectrum disorder: a systematic review and Meta-analysis. Nutrients 12:792. doi: 10.3390/nu12030792, 32192218 PMC7146354

[ref46] Ingram N. T. Daniels C. Riggins N. Stevane J. R. Staats P. S. (2026). Without getting under your skin: non-invasive stimulation activates the vagus nerve. Front. Neurosci. 20:1829474. doi: 10.3389/fnins.2026.1829474, 42359347 PMC13290870

[ref47] Ismail F. Y. Shapiro B. K. (2019). What are neurodevelopmental disorders? Curr. Opin. Neurol. 32, 611–616. doi: 10.1097/WCO.0000000000000710, 31116115

[ref48] Jenkins D. D. Khodaparast N. O’Leary G. H. Washburn S. N. Covalin A. Badran B. W. (2021). Transcutaneous auricular neurostimulation (tAN): a novel adjuvant treatment in neonatal opioid withdrawal syndrome. Front. Hum. Neurosci. 15:648556. doi: 10.3389/fnhum.2021.648556, 33762918 PMC7982745

[ref49] Jenkins D. D. Moss H. G. Adams L. E. Hunt S. Dancy M. Huffman S. M. . (2023). Higher dose noninvasive transcutaneous auricular Vagus nerve stimulation increases feeding volumes and white matter microstructural complexity in open-label study of infants slated for gastrostomy tube. J. Pediatr. 262:113563. doi: 10.1016/j.jpeds.2023.113563, 37329979 PMC11000235

[ref50] Jiakai H. E. Jinling Z. Yu W. Shaoyuan L. I. Jiliang F. Shuai Z. . (2023). Transcutaneous auricular vagus nerve stimulation would be an alternative to implantable cervical vagus nerve stimulation in some situation. Journal of Traditional Chinese Medicine = Chung I Tsa Chih Ying Wen Pan 43, 627–630. doi: 10.19852/j.cnki.jtcm.20230308.002, 37147767 PMC10133952

[ref51] Kaelberer M. M. Buchanan K. L. Klein M. E. Barth B. B. Montoya M. M. Shen X. . (2018). A gut-brain neural circuit for nutrient sensory transduction. Science 361:eaat5236. doi: 10.1126/science.aat5236, 30237325 PMC6417812

[ref52] Kalfiřt L. Su C.-T. Fu C.-P. Lee S.-D. Yang A.-L. (2023). Motor skills, heart rate variability, and arterial stiffness in children with autism Spectrum disorder. Healthcare 11:1898. doi: 10.3390/healthcare11131898, 37444732 PMC10341336

[ref53] Kang D.-W. Park J. G. Ilhan Z. E. Wallstrom G. LaBaer J. Adams J. B. . (2013). Reduced incidence of Prevotella and other fermenters in intestinal microflora of autistic children. PLoS One 8:e68322. doi: 10.1371/journal.pone.0068322, 23844187 PMC3700858

[ref54] Khuanjing T. Maneechote C. Ongnok B. Prathumsap N. Arinno A. Chunchai T. . (2023). Vagus nerve stimulation and acetylcholinesterase inhibitor donepezil provide cardioprotection against trastuzumab-induced cardiotoxicity in rats by attenuating mitochondrial dysfunction. Biochem. Pharmacol. 217:115836. doi: 10.1016/j.bcp.2023.115836, 37816466

[ref55] Kim J. S. Kirkland R. A. Lee S. H. Cawthon C. R. Rzepka K. W. Minaya D. M. . (2020). Gut microbiota composition modulates inflammation and structure of the vagal afferent pathway. Physiol. Behav. 225:113082. doi: 10.1016/j.physbeh.2020.113082, 32682966 PMC7484462

[ref56] Kim W.-J. Lee Y.-S. Hong K. H. Choi H. Song J.-J. Hwang H.-J. (2025). Effect of transcutaneous auricular vagus nerve stimulation on stress regulation: an EEG and questionnaire study. Front. Digital Health 7:1593614. doi: 10.3389/fdgth.2025.1593614, 40958978 PMC12434109

[ref57] Kim S. Park I. Lee J. H. Kim S. Jang D.-H. Jo Y. H. (2022). Vagus nerve stimulation improves mitochondrial dysfunction in post–cardiac arrest syndrome in the Asphyxial cardiac arrest model in rats. Front. Neurosci. 16:762007. doi: 10.3389/fnins.2022.762007, 35692415 PMC9178208

[ref58] Lerman I. Hauger R. Sorkin L. Proudfoot J. Davis B. Huang A. . (2016). Noninvasive transcutaneous vagus nerve stimulation decreases whole blood culture-derived cytokines and chemokines: a randomized, blinded, healthy control pilot trial. Neuromodulation 19, 283–291. doi: 10.1111/ner.12398 26990318

[ref59] Levy M. L. Levy K. M. Hoff D. Amar A. P. Park M. S. Conklin J. M. . (2010). Vagus nerve stimulation therapy in patients with autism spectrum disorder and intractable epilepsy: results from the vagus nerve stimulation therapy patient outcome registry: clinical article. J. Neurosurg. Pediatr. 5, 595–602. doi: 10.3171/2010.3.PEDS09153, 20515333

[ref60] Lewandowska-Pietruszka Z. Figlerowicz M. Mazur-Melewska K. (2023). Microbiota in autism Spectrum disorder: a systematic review. Int. J. Mol. Sci. 24:16660. doi: 10.3390/ijms242316660, 38068995 PMC10706819

[ref61] Liang J. Li X. Mao C. Qin Z. Wang R. (2026). Electro-microbial interplay in the gut–brain axis: an emerging paradigm for gut microbiota modulation via vagus nerve stimulation. Curr. Opin. Pharmacol. 86:102588. doi: 10.1016/j.coph.2025.102588, 41421301

[ref62] Liu J. Dai Q. Qu T. Ma J. Lv C. Wang H. . (2024). Ameliorating effects of transcutaneous auricular vagus nerve stimulation on a mouse model of constipation-predominant irritable bowel syndrome. Neurobiol. Dis. 193:106440. doi: 10.1016/j.nbd.2024.106440, 38369213

[ref63] Lord C. Brugha T. S. Charman T. Cusack J. Dumas G. Frazier T. . (2020). Autism spectrum disorder. Nat. Rev. Dis. Prim. 6:5. doi: 10.1038/s41572-019-0138-4, 31949163 PMC8900942

[ref64] Luna F. G. Lupiáñez J. König S. Garscha U. Fischer R. (2025). Can transcutaneous auricular vagus nerve stimulation mitigate vigilance loss? Examining the effects of stimulation at individualized versus constant current intensity. Psychophysiology 62:e14670. doi: 10.1111/psyp.14670, 39169561 PMC11775886

[ref65] Ma B. Liang J. Dai M. Wang J. Luo J. Zhang Z. . (2019). Altered gut microbiota in Chinese children with autism Spectrum disorders. Front. Cell. Infect. Microbiol. 9:40. doi: 10.3389/fcimb.2019.00040, 30895172 PMC6414714

[ref66] Machetanz K. Berelidze L. Guggenberger R. Gharabaghi A. (2021). Transcutaneous auricular vagus nerve stimulation and heart rate variability: analysis of parameters and targets. Auton. Neurosci. 236:102894. doi: 10.1016/j.autneu.2021.102894, 34662844

[ref67] Mamun A. A. Geng P. Wang S. Shao C. Xiao J. (2025). IUPHAR review: targeted therapies of signaling pathways based on the gut microbiome in autism spectrum disorders: mechanistic and therapeutic applications. Pharmacol. Res. 211:107559. doi: 10.1016/j.phrs.2024.107559, 39733842

[ref68] Manning K. E. McAllister C. J. Ring H. A. Finer N. Kelly C. L. Sylvester K. P. . (2016). Novel insights into maladaptive behaviours in Prader–Willi syndrome: serendipitous findings from an open trial of vagus nerve stimulation. J. Intellect. Disabil. Res. 60, 149–155. doi: 10.1111/jir.12203, 26018613 PMC4950305

[ref69] Manta S. El Mansari M. Debonnel G. Blier P. (2013). Electrophysiological and neurochemical effects of long-term vagus nerve stimulation on the rat monoaminergic systems. Int. J. Neuropsychopharmacol. 16, 459–470. doi: 10.1017/S1461145712000387, 22717062

[ref70] Marano G. Sfratta G. Marzo E. M. Cozzo G. Abate F. Traversi G. . (2025). The pediatric microbiota–gut–brain Axis: implications for neuropsychiatric development and intervention. Children 12:1561. doi: 10.3390/children12111561, 41300676 PMC12651788

[ref71] Marvin M. Gardner F. Sarsfield K. Travagli R. Doheny K. (2019). Increased frequency of skin-to-skin contact is associated with enhanced vagal tone and improved health outcomes in preterm neonates. Am. J. Perinatol. 36, 505–510. doi: 10.1055/s-0038-1669946, 30193382 PMC6405324

[ref72] Mayer E. A. Tillisch K. Gupta A. (2015). Gut/brain axis and the microbiota. J. Clin. Invest. 125, 926–938. doi: 10.1172/JCI76304, 25689247 PMC4362231

[ref73] Minaya D. M. Kim J. S. Kirkland R. Allen J. Cullinan S. Maclang N. . (2024). Transfer of microbiota from lean donors in combination with prebiotics prevents excessive weight gain and improves gut-brain vagal signaling in obese rats. Gut Microbes 16:2421581. doi: 10.1080/19490976.2024.2421581, 39485288 PMC11540078

[ref74] Morais L. H. Schreiber H. L. Mazmanian S. K. (2021). The gut microbiota–brain axis in behaviour and brain disorders. Nat. Rev. Microbiol. 19, 241–255. doi: 10.1038/s41579-020-00460-0, 33093662

[ref75] Mörkl S. Narrath M. Schlotmann D. Sallmutter M.-T. Putz J. Lang J. . (2025). Multi-species probiotic supplement enhances vagal nerve function – results of a randomized controlled trial in patients with depression and healthy controls. Gut Microbes 17:2492377. doi: 10.1080/19490976.2025.2492377 40298641 PMC12045568

[ref76] Morris-Rosendahl D. J. Crocq M.-A. (2020). Neurodevelopmental disorders—the history and future of a diagnostic T2concept. Dialogues Clin. Neurosci. 22, 65–72. doi: 10.31887/DCNS.2020.22.1/macrocq 32699506 PMC7365295

[ref77] Novau-Ferré N. Papandreou C. Rojo-Marticella M. Canals-Sans J. Bulló M. (2025). Gut microbiome differences in children with attention deficit hyperactivity disorder and autism Spectrum disorder and effects of probiotic supplementation: a randomized controlled trial. Res. Dev. Disabil. 161:105003. doi: 10.1016/j.ridd.2025.105003, 40184961

[ref78] Olson C. A. Vuong H. E. Yano J. M. Liang Q. Y. Nusbaum D. J. Hsiao E. Y. (2018). The gut microbiota mediates the anti-seizure effects of the ketogenic diet. Cell 174:497. doi: 10.1016/j.cell.2018.06.051, 30007420 PMC6062008

[ref79] Owens M. M. Jacquemet V. Napadow V. Lewis N. Beaumont E. (2024). Brainstem neuronal responses to transcutaneous auricular and cervical vagus nerve stimulation in rats. J. Physiol. 602, 4027–4052. doi: 10.1113/JP286680, 39031516 PMC11326965

[ref80] Parolisi S. Montanari C. Borghi E. Cazzorla C. Zuvadelli J. Tosi M. . (2023). Possible role of tryptophan metabolism along the microbiota-gut-brain axis on cognitive & behavioral aspects in phenylketonuria. Pharmacol. Res. 197:106952. doi: 10.1016/j.phrs.2023.106952, 37804926

[ref81] Pavlov V. A. Tracey K. J. (2012). The vagus nerve and the inflammatory reflex—linking immunity and metabolism. Nat. Rev. Endocrinol. 8, 743–754. doi: 10.1038/nrendo.2012.189, 23169440 PMC4082307

[ref82] Pulikkan J. Maji A. Dhakan D. B. Saxena R. Mohan B. Anto M. M. . (2018). Gut microbial Dysbiosis in Indian children with autism Spectrum disorders. Microb. Ecol. 76, 1102–1114. doi: 10.1007/s00248-018-1176-2, 29564487

[ref83] Quan Y. Zhou T. (2026). Efficacy and mechanisms of vagus nerve stimulation in irritable bowel syndrome: a comprehensive literature review. Front. Immunol. 17:1769070. doi: 10.3389/fimmu.2026.1769070, 41890751 PMC13013011

[ref84] Ramirez J. Guarner F. Bustos Fernandez L. Maruy A. Sdepanian V. L. Cohen H. (2020). Antibiotics as major disruptors of gut microbiota. Front. Cell. Infect. Microbiol. 10:572912. doi: 10.3389/fcimb.2020.572912, 33330122 PMC7732679

[ref85] Rei D. Saha S. Haddad M. Rubio A. H. Perlaza B. L. Berard M. . (2022). Age-associated gut microbiota impairs hippocampus-dependent memory in a vagus-dependent manner. JCI Insight 7:e147700. doi: 10.1172/jci.insight.147700, 35737457 PMC9462480

[ref86] Rubas N. C. Torres A. Maunakea A. K. (2025). The gut microbiome and Epigenomic reprogramming: mechanisms, interactions, and implications for human health and disease. Int. J. Mol. Sci. 26:8658. doi: 10.3390/ijms26178658, 40943575 PMC12429217

[ref87] Sanders T. H. Weiss J. Hogewood L. Chen L. Paton C. McMahan R. L. . (2019). Cognition-enhancing Vagus nerve stimulation alters the epigenetic landscape. J. Neurosci. 39, 2407–2418. doi: 10.1523/JNEUROSCI.2407-18.2019, 30804093 PMC6495123

[ref88] Schmausser M. Holland A. Beresford-Webb J. Eglen S. J. Manning K. Aman L. . (2024). Effects of long-term transcutaneous auricular vagus nerve stimulation on circadian vagal activity in people with Prader-Willi syndrome: a case-series. Res. Dev. Disabil. 154:104855. doi: 10.1016/j.ridd.2024.104855, 39405838

[ref89] Sharma B. Agriantonis G. Twelker K. Ebelle D. Kiernan S. Siddiqui M. . (2025). Gut microbiota serves as a crucial independent biomarker in inflammatory bowel disease (IBD). Int. J. Mol. Sci. 26:2503. doi: 10.3390/ijms26062503, 40141145 PMC11942158

[ref90] Sharon G. Cruz N. J. Kang D.-W. Gandal M. J. Wang B. Kim Y.-M. . (2019). Human gut microbiota from autism Spectrum disorder promote behavioral symptoms in mice. Cell 177, 1600–1618.e17. doi: 10.1016/j.cell.2019.05.004, 31150625 PMC6993574

[ref91] Sigrist C. Torki B. Bolz L.-O. Jeglorz T. Bolz A. Koenig J. (2023). Transcutaneous auricular vagus nerve stimulation in pediatric patients: a systematic review of clinical treatment protocols and stimulation parameters. Neuromodulation 26, 507–517. doi: 10.1016/j.neurom.2022.07.007, 35995653

[ref92] Slykerman R. F. Thompson J. Waldie K. E. Murphy R. Wall C. Mitchell E. A. (2017). Antibiotics in the first year of life and subsequent neurocognitive outcomes. Acta Paediatr. 106, 87–94. doi: 10.1111/apa.13613, 27701771

[ref93] Spiller A. L. Costa B. G. D. Yoshihara R. N. Y. Nogueira E. J. Z. Castelhano N. S. Santos A. . (2025). Ultra-processed foods, gut microbiota, and inflammatory bowel disease: a critical review of emerging evidence. Nutrients 17:2677. doi: 10.3390/nu17162677, 40871705 PMC12389707

[ref94] Strati F. Cavalieri D. Albanese D. De Felice C. Donati C. Hayek J. . (2017). New evidences on the altered gut microbiota in autism spectrum disorders. Microbiome 5:24. doi: 10.1186/s40168-017-0242-1, 28222761 PMC5320696

[ref95] Sukmajaya A. C. Lusida M. I. Soetjipto Setiawati Y. (2021). Systematic review of gut microbiota and attention-deficit hyperactivity disorder (ADHD). Ann. General Psychiatry 20:12. doi: 10.1186/s12991-021-00330-w PMC7888126 33593384

[ref96] Sun K. Li Y. Zhai Z. Yin H. Liang S. Zhai F. . (2024). Effects of transcutaneous auricular vagus nerve stimulation and exploration of brain network mechanisms in children with high−functioning autism spectrum disorder: study protocol for a randomized controlled trial. Front. Psych. 15:1337101. doi: 10.3389/fpsyt.2024.1337101, 38374975 PMC10875019

[ref97] Talat A. Zuberi A. Khan A. U. (2025). Unravelling the gut–microbiome–brain Axis: implications for infant neurodevelopment and future therapeutics. Curr. Microbiol. 82:390. doi: 10.1007/s00284-025-04370-3, 40670809

[ref98] Tan Q. Orsso C. E. Deehan E. C. Kung J. Y. Tun H. M. Wine E. . (2021). Probiotics, prebiotics, synbiotics, and fecal microbiota transplantation in the treatment of behavioral symptoms of autism spectrum disorder: a systematic review. Autism Res. 14, 1820–1836. doi: 10.1002/aur.2560, 34173726

[ref99] Thapar A. Cooper M. (2016). Attention deficit hyperactivity disorder. Lancet 387, 1240–1250. doi: 10.1016/S0140-6736(15)00238-X, 26386541

[ref100] Thomaidi S. Sarantaki A. Tzitiridou Chatzopoulou M. Orovou E. Jotautis V. Papoutsis D. (2025). The rising global cesarean section rates and their impact on maternal and child health: a scoping review. J. Clin. Med. 14:8102. doi: 10.3390/jcm14228102, 41303138 PMC12653877

[ref101] Tracey K. J. (2002). The inflammatory reflex. Nature 420, 853–859. doi: 10.1038/nature01321, 12490958

[ref102] Tyler W. J. (2025). Transcutaneous auricular vagus nerve stimulation for treating emotional dysregulation and inflammation in common neuropsychiatric disorders. Brain Sci. 16:8. doi: 10.3390/brainsci16010008, 41594729 PMC12838753

[ref103] Van Hoorn A. Carpenter T. Oak K. Laugharne R. Ring H. Shankar R. (2019). Neuromodulation of autism spectrum disorders using vagal nerve stimulation. J. Clin. Neurosci. 63, 8–12. doi: 10.1016/j.jocn.2019.01.042, 30732986

[ref104] Wakefield C. Courchesne M. Nygard K. Frasch M. G. (2025). “Microglial and macrophage plasticity and regional cerebral blood flow in the prenatal brain and gut under vagus nerve stimulation,” in Psychoneuroimmunology, ed. Yan Q. (New York, NY: Springer), 285–301. 10.1007/978-1-0716-4200-9_15 39546236

[ref105] Wang L. Conlon M. A. Christophersen C. T. Sorich M. J. Angley M. T. (2014). Gastrointestinal microbiota and metabolite biomarkers in children with autism Spectrum disorders. Biomark. Med 8, 331–344. doi: 10.2217/bmm.14.12, 24712423

[ref106] Wang Y. Tan Q. Pan M. Yu J. Wu S. Tu W. . (2024). Minimally invasive vagus nerve stimulation modulates mast cell degranulation via the microbiota-gut-brain axis to ameliorate blood-brain barrier and intestinal barrier damage following ischemic stroke. Int. Immunopharmacol. 132:112030. doi: 10.1016/j.intimp.2024.112030, 38603861

[ref107] Wang Z. Yuan X. Zhang Q. Wen J. Cheng T. Qin X. . (2022). Effects of stable Vagus nerve stimulation efficacy on autistic behaviors in ten pediatric patients with drug resistant epilepsy: an observational study. Front. Pediatr. 10:846301. doi: 10.3389/fped.2022.846301, 35311037 PMC8924444

[ref108] Wang Y. Zhu J. Zou N. Zhang L. Wang Y. Zhang M. . (2023). Pathogenesis from the microbial-gut-brain axis in white matter injury in preterm infants: a review. Front. Integr. Neurosci. 17:1051689. doi: 10.3389/fnint.2023.1051689, 37006416 PMC10060642

[ref109] Xue R. Sun L. Yu X. Li D. Zang W. (2017). Vagal nerve stimulation improves mitochondrial dynamics via an M_3_ receptor/ca MKK β/ AMPK pathway in isoproterenol-induced myocardial ischaemia. J. Cell. Mol. Med. 21, 58–71. doi: 10.1111/jcmm.12938, 27491814 PMC5192749

[ref110] Yang C. Xiao H. Zhu H. Du Y. Wang L. (2024). Revealing the gut microbiome mystery: a meta-analysis revealing differences between individuals with autism spectrum disorder and neurotypical children. Biosci. Trends 18, 233–249. doi: 10.5582/bst.2024.01123, 38897955

[ref111] Yuan H. Silberstein S. D. (2017). Vagus nerve stimulation and headache. Headache 57, 29–33. doi: 10.1111/head.12721, 26473407

[ref112] Yue Y. Zou L. Li H. Xia Y. Ren Z. Yang F. . (2023). Therapeutic effect of implanted and non-invasive vagus nerve stimulation on heroin-induced anxiety. Biochem. Biophys. Res. Commun. 652, 46–54. doi: 10.1016/j.bbrc.2023.02.041, 36809704

[ref113] Zaehle Krauel (2021). “Transcutaneous vagus nerve stimulation in patients with attention-deficit/hyperactivity disorder: a viable option?” in Progress in Brain Research, eds. Kadosh R. C. Zaehle T. Krauel K., vol. 264 (Amsterdam: Elsevier), 171–190. 10.1016/bs.pbr.2021.03.001 34167655

[ref114] Zhang Y. Li X. Lu S. Guo H. Zhang Z. Zheng H. . (2024). Stress triggers gut dysbiosis via CRH-CRHR1-mitochondria pathway. Npj Biofilms and Microbiomes 10:93. doi: 10.1038/s41522-024-00571-z, 39349483 PMC11442948

[ref115] Zhao J. Lei Y. Mu C. Wu Y. Fang R. Wu D. . (2025). taVNS alleviates preeclampsia-induced vascular endothelial dysfunction via α7nAChR- IP3R1/GRP75/VDAC1 signal pathway. Inflamm. Res. 74:133. doi: 10.1007/s00011-025-02100-w, 40986011

[ref116] Zhi J. Zhang S. Huang M. Qin H. Xu H. Chang Q. . (2024). Transcutaneous auricular vagus nerve stimulation as a potential therapy for attention deficit hyperactivity disorder: modulation of the noradrenergic pathway in the prefrontal lobe. Front. Neurosci. 18:1494272. doi: 10.3389/fnins.2024.1494272, 39697776 PMC11652481

